# Molecular and Cellular Basis of Autosomal Recessive Primary Microcephaly

**DOI:** 10.1155/2014/547986

**Published:** 2014-12-08

**Authors:** Marine Barbelanne, William Y. Tsang

**Affiliations:** ^1^Institut de Recherches Cliniques de Montréal, 110 avenue des Pins Ouest, Montréal, QC, Canada H2W 1R7; ^2^Faculté de Médecine, Université de Montréal, Montréal, QC, Canada H3C 3J7; ^3^Division of Experimental Medicine, McGill University, Montréal, QC, Canada H3A 1A3

## Abstract

Autosomal recessive primary microcephaly (MCPH) is a rare hereditary neurodevelopmental disorder characterized by a marked reduction in brain size and intellectual disability. MCPH is genetically heterogeneous and can exhibit additional clinical features that overlap with related disorders including Seckel syndrome, Meier-Gorlin syndrome, and microcephalic osteodysplastic dwarfism. In this review, we discuss the key proteins mutated in MCPH. To date, MCPH-causing mutations have been identified in twelve different genes, many of which encode proteins that are involved in cell cycle regulation or are present at the centrosome, an organelle crucial for mitotic spindle assembly and cell division. We highlight recent findings on MCPH proteins with regard to their role in cell cycle progression, centrosome function, and early brain development.

## 1. Introduction

Autosomal recessive primary microcephaly (MCPH) is a rare condition associated with developmental anomaly of the brain. This neurodevelopmental disorder is characterized by a reduced occipitofrontal head circumference (OFC) at birth to at least 2-3 standard deviations below the mean for sex, age, and ethnicity, a slower than average growth in OFC after birth, and prenatal onset as early as the second trimester of gestation [1–6]. MCPH patients possess a small brain with simplified gyri and exhibit varying degrees of intellectual disability; however, the architecture of the brain in general is not grossly affected. In some instances, MCPH is associated with additional clinical features, including short stature, mild seizures, or skeletal abnormalities, and shows genetic and clinical overlap with related disorders such as Seckel syndrome (SCKL; OMIM 210600, 606744, 608664, 613676, 613823, 614728, 614851, 615807), Meier-Gorlin syndrome (OMIM 224690, 613800, 613804), and microcephalic osteodysplastic dwarfism (OMIM 210710, 210720, 210730) [7–12]. Although MCPH was traditionally distinguished from other disorders by height, short stature is no longer a distinguishing feature. Furthermore, it is now known that mutations in the same gene can cause MCPH and SCKL. In light of these observations, it is tempting to speculate that there must be a considerable overlap between the pathological mechanisms underlying MCPH and related disorders.

## 2. MCPH Loci and Brain Development

To date, twelve genetic loci (MCPH-MCPH12) are implicated in MCPH (Table 1). The majority of mutations reported in these genes are frameshift or nonsense mutations leading to truncated proteins that are nonfunctional (please refer to references in Table 1). Perhaps not surprisingly, MCPH gene products are shown to be highly expressed in neuroepithelial or neuroprogenitor cells during early brain development [13–17]. Brain size at birth is primarily dependent on the ability of neuroprogenitor cells to proliferate and self-renew [9, 10, 18, 19]. While symmetrical division of a neuroprogenitor cell results in the generation of two identical neuroprogenitor cells (thereby increasing the progenitor pool), asymmetrical division leads to the production of one progenitor cell (thereby maintaining the progenitor pool) and a committed precursor, which eventually undergoes migration and differentiates into neurons [20, 21]. Conceivably, any perturbation that upsets the balance between symmetric and asymmetric division can drastically reduce the number of neuroprogenitor and neuronal cells, leading to reduced brain size [10]. Although such a mechanism is appealing, it is important to note that additional mechanisms, including cell proliferation defects, enhanced cell death/apoptosis, abnormal neuronal migration and/or differentiation, can also impair brain development and contribute to the development of MCPH [10]. Interestingly, a significant number of MCPH proteins identified thus far are found to be associated with the centrosome [15, 16, 22–29], an organelle intimately connected with cell division, suggesting that proper cell cycle control could play an important role in neurogenesis.

## 3. Centrosome Structure and Function

**Figure 1:**
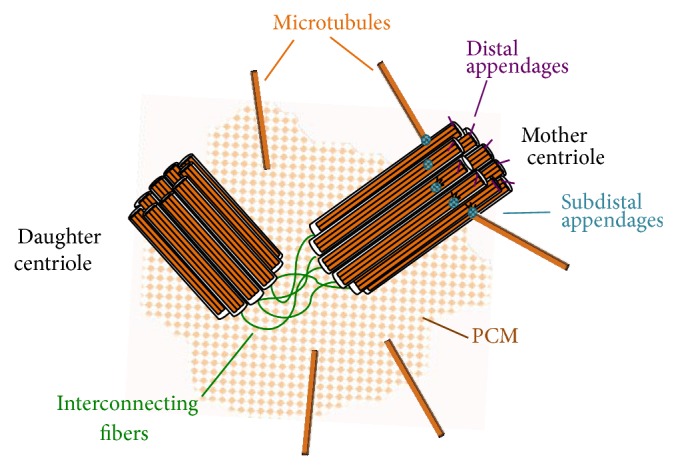
Centrosome structure. Centrosomes are small organelles composed of two perpendicular centrioles (orange cylinders), a mother and a daughter, linked together by interconnecting fibres (dark green). The centrioles are surrounded by an amorphous pericentriolar matrix (dotted orange background) involved in the nucleation and anchoring of cytoplasmic microtubules. Contrary to the daughter centriole, the mother centriole possesses distal (purple) and subdistal (blue) appendages necessary for cilia assembly and microtubule anchoring, respectively.

The centrosome is the major microtubule-organizing center in mammalian cells and modulates diverse cellular processes such as cell cycle progression, cell shape, polarity, adhesion and motility, cilia assembly, DNA damage response, intracellular transport, positioning of cellular organelles, mitotic spindle formation, positioning and orientation, and genome stability [30–35]. This organelle is composed of a pair of centrioles, a mother and a daughter, surrounded by an amorphous pericentriolar matrix (PCM) (Figure 1). Centrioles, cylindrical structures consisting of nine triplets of stabilized microtubules, organize the PCM, which in turn nucleates and anchors cytoplasmic microtubules necessary for mitotic spindle assembly and chromosome segregation. Centrosome number, morphology, and function are tightly regulated during the cell cycle [36–39]. A cell in the G1 phase has one centrosome. Centrosome duplication occurs once in the S phase and entails the synthesis of two new centrioles or procentrioles adjacent to the existing centrioles. At the G2/M transition, the duplicated centrosomes separate and migrate to opposite poles of the cell, and through a process known as centrosome maturation, additional proteins are recruited to the PCM to increase its microtubule-nucleating and -anchoring capacity essential for cell division. Defects in centrosome duplication and/or maturation are known to compromise cell cycle progression and cell division, resulting in aneuploidy, cell cycle arrest, cell death, and/or uncontrolled cell growth. Indeed, centrosome dysfunction has been linked to a wide variety of human diseases including MCPH, but how exactly does it impede the cell cycle and affect brain development at the mechanistic level? In the next section, we will highlight the current status of our knowledge on the role of each MCPH gene product in cell cycle regulation, centrosome function, and neurogenesis.

## 4. MICROCEPHALIN

*MCPH1/MICROCEPHALIN* is the first disease gene identified for MCPH and it encodes MICROCEPHALIN, a multifunctional protein that participates in various cellular processes [13, 40, 41]. MICROCEPHALIN possesses three BRCT (BRCA1 C-terminal) domains commonly found in proteins involved in cell cycle control, DNA damage response, and DNA repair [42]. Indeed, it functions to recruit the chromatin remodelling complex SWI-SNF (switch/sucrose nonfermentable) to DNA lesions and interacts with the E2F1 transcription factor to regulate genes involved in DNA repair and apoptosis [43–45]. MICROCEPHALIN also associates with CONDENSIN II, a protein involved in chromosome condensation, perhaps explaining why a loss of MICROCEPHALIN function triggers early cell cycle progression and premature chromosome condensation [46, 47]. Besides its nuclear localization, MICROCEPHALIN also localizes to the centrosome throughout the cell cycle and interacts with PERICENTRIN, a PCM component critical for centrosome maturation, to control the localization of CHK1 (checkpoint kinase 1) to the centrosome [22, 48, 49]. In the absence of MICROCEPHALIN, CHK1 is mislocalized and cannot phosphorylate and inactivate CDC25B (cell division cycle 25B), thereby triggering premature CDK1 (cyclin-dependent kinase 1) activation and early mitotic entry. Interestingly, PERICENTRIN is also mislocalized from the centrosome in the absence of MICROCEPHALIN, suggesting that the latter recruits the former to the centrosome. Although two mouse models of* Mcph1* show no obvious brain phenotype [50, 51], a conditional knock-out causes untimely entry into mitosis, mitotic spindle misorientation, and a premature switch of neuroprogenitors from symmetric to asymmetric division, resulting in primary microcephaly (Table 2) [49, 52]. Interestingly, silencing* Cdc25b* is sufficient to rescue these phenotypes, suggesting that proper mitotic entry and progression are needed to maintain a balance between neuroprogenitor proliferation and neuronal differentiation.

## 5. WDR62 (WD Repeat-Containing Protein 62)

*WDR62* is the second most frequently mutated gene in MCPH, accounting for about 10% of cases [16, 23, 53, 54]. Its encoded protein product possesses several WD40 (beta-transducin repeat) domains that mediate protein-protein interactions. WDR62 is predominantly a nuclear protein during interphase and accumulates at the spindle poles during mitosis [16, 55, 56]. The primary function of WDR62 is to preserve centrosome/spindle pole integrity after bipolar spindle formation, since a loss of this protein leads to the dispersal of PCM components PERICENTRIN, *γ*-TUBULIN, and CDK5RAP2 (CDK5 regulatory subunit-associated protein 2) (these three PCM proteins are known to interact with each other) from metaphase centrosomes [55, 56]. In addition, WDR62 is a substrate of c-Jun N-terminal kinase (JNK), active at the centrosome during mitosis, and phosphorylation of WDR62 by JNK is required for mitotic spindle organization [55, 57]. Depletion of either Wdr62 (shRNA knock-down, Table 2) or Jnk induces spindle misorientation and triggers the asymmetric division of neuroprogenitors in the rat telencephalon, leading to their premature differentiation into neurons [58]. In another study, morpholino-mediated knock-down of wdr62 or two other microcephaly proteins, aspm (abnormal spindle-like microcephaly-associated protein) and sil (*STIL* in human; SCL/TAL1-interrupting locus), in zebrafish causes a significant reduction in head and eye size [59]. This phenotype is believed to be due to a failure in metaphase progression, leading to increased cell death [59]. Likewise, neuroprogenitor cells of mice deficient in Wdr62 exhibit spindle assembly checkpoint activation, delayed mitotic progression, and cell death, resulting in reduced brain size (Table 2) [60]. Finally, lymphoblastoid cells derived from patients with compound heterozygous mutations in* WDR62* exhibit mitotic spindle defects as well as abnormal centrosomal protein localization [56]. Mechanistically, WDR62 physically and genetically interacts with AURORA A [60], a serine/threonine protein kinase that controls centrosome maturation, spindle formation, and mitotic progression. AURORA A is a PCM protein, and its targeting to and subsequent activation at the mitotic spindle are dependent on another centrosomal protein CEP192 (centrosomal protein of 192 kDa) (discussed later) [61]. Thus, WDR62 appears to control the fate of neuroprogenitors cells partially through AURORA A.

## 6. CDK5RAP2 (CDK5 Regulatory Subunit-Associated Protein 2)

By virtue of its ability to interact with *γ*-TUBULIN, CDK5RAP2 is a PCM protein crucial for microtubule nucleation [62, 63]. Depletion of CDK5RAP2 delocalizes *γ*-TUBULIN from centrosomes, thereby preventing centrosomal microtubule formation. Another PCM component, PERICENTRIN, also physically interacts with CDK5RAP2, and recruitment of the latter to the centrosome depends on the former but not vice versa [64–66]. In addition to its role in PCM regulation, CDK5RAP2 is also required for centriole and centrosome cohesion, and ablation of this protein induces unscheduled centriole splitting, leading to amplified centrosomes and multipolar spindles [67, 68]. Furthermore, CDK5RAP2 has the capacity to bind DNA and functions as a transcription activator to regulate the expression of two spindle checkpoint genes,* BUBR1* (budding uninhibited by benzimidazole-related 1) and* MAD2* (mitotic arrest deficient 2) [69]. Recently, the role of Cdk5rap2 in neurogenesis was examined in mice. Consistent with the functional relationship between Cdk5rap2 and Pericentrin, knock-down of either protein depletes the neural progenitor pool and triggers cell cycle exit, leading to premature neuronal differentiation without substantial apoptosis in the developing mouse neocortex of an in utero electroporation model (Table 2) [70]. In contrast, a different study showed that although the* Hertwig's anemia* mouse exhibits microcephaly, most neuroprogenitor cells undergo apoptosis instead of differentiating into neurons after exiting the cell cycle abruptly (Table 2) [71]. Thus, CDK5RAP2 could play a role in neuroprogenitor cell death and neuronal differentiation.

## 7. CASC5 (Cancer Susceptibility Candidate 5)

*CASC5* is among the most recently identified genes responsible for MCPH [72]. Unlike most MCPH proteins which localize to centrosomes, CASC5 is a kinetochore scaffold protein required for the proper attachment of chromatin to the mitotic apparatus [73]. It also associates with BUB1 (budding uninhibited by benzimidazoles 1) and BUBR1 to control the spindle assembly checkpoint. Depletion of this protein induces chromosome misalignment and accelerates entry into mitosis, a phenotype reminiscent of MICROCEPHALIN loss [74–77]. Future experiments, including the use of animal models, are needed to further delineate the molecular and cellular function of CASC5 and to define its role in neurogenesis.

## 8. ASPM (Abnormal Spindle-Like Microcephaly-Associated Protein)

Mutations in the* ASPM* gene constitute the most common cause of MCPH and accounts for about 25–50% of cases [6, 10, 14, 78–90]. ASPM contains a microtubule-binding domain, two calponin homology domains commonly found in cytoskeletal proteins, and multiple IQ calmodulin-binding motifs. This protein may be required for the maintenance of centrosome/spindle integrity because, like WDR62, it is mostly concentrated in the nucleus and only relocates to the spindle pole during mitosis [24]. Furthermore, CALMODULIN, a calcium-binding protein known to interact with PERICENTRIN and ASPM, also exhibits strong staining at the spindle poles [91]. Localization of ASPM to the spindle pole is greatly diminished in fibroblasts derived from a patient carrying a homozygous* ASPM* mutation [92]. Depletion of ASPM in human cells affects spindle positioning and alters the division symmetry from symmetrical to asymmetrical, leading to cytokinesis failure [92]. Similarly, ablation of Aspm enhances asymmetric cell division and premature differentiation of mouse telencephalic neuroprogenitor cells without causing cell cycle arrest (Table 2) [93]. Likewise,* aspm* mutant flies display spindle-positioning defects, in addition to increased apoptosis [94, 95]. In contrast, mutant mice (*Aspm* ^*1-25*^ and* Aspm* ^*1-7*^) expressing truncated proteins show no major alteration of cleavage plane orientation but are still microcephalic [96]. Taken together, ASPM may have additional function besides spindle positioning and division axis orientation critical for symmetric cell division. Of note, although* Aspm* mutant mice and flies exhibit microcephaly, these animals also have impaired fertility due to a massive loss of germ cells [96–99]. These observations, coupled with findings that ASPM and MICROCEPHALIN are highly upregulated in different types of cancer [100–103], suggest that these two proteins could also positively regulate cell proliferation in multiple cell types.

## 9. CENPJ (Centromere Protein J)

A handful of core centrosomal components, including four microcephaly proteins CENPJ, STIL, CEP135 (centrosomal protein of 135 kDa), and CEP152 (centrosomal protein of 152 kDa), were recently identified as essential regulators of centriole duplication [104–106]. Centriole duplication is thought to occur in several sequential steps, wherein CEP152 and CEP192 first interact with each other to recruit polo-like kinase 4 (PLK4) to the site of centriole assembly [107–111]. This event is followed by the recruitment of SAS-6 (spindle assembly abnormal protein 6 homolog) and STIL, proteins that dictate the nine-fold radial symmetric arrangement of microtubules in centrioles, and finally CENPJ, to new centrioles [106, 112–117]. CENPJ is known to interact with STIL, CEP135, and CEP152, and in addition, possesses the capacity to bind microtubules and to associate with CEP120 (centrosomal protein of 120 kDa) and SPICE1 (spindle and centriole associated protein 1), two proteins essential for centriole elongation [110, 111, 118–123]. Indeed, CENPJ is specifically involved in the elongation step of centriole duplication, and depletion of this protein leads to the formation of nascent centrioles that fail to reach full length, whereas overexpression promotes the formation of elongated centrioles [124–126]. Both moderate and excessive centriole elongations are detrimental to cells, causing loss of centrosome integrity and formation of multipolar spindles [126, 127]. In addition to its role in centriole biogenesis, CENPJ also interacts with several PCM components to regulate the size of the PCM [128–130]. CENPJ was also found to play an important role in controlling the prefrontal cortex development in human [131]. Targeted inactivation of* Cenpj* in mice recapitulates many of the clinical features of MCPH and SCKL, including mitotic failure and massive cell death during embryonic development (Table 2) [132]. Notably, of the 12 MCPH proteins identified to date, only deficiencies in CENPJ and CEP152 are known to cause both MCPH and SCKL, the latter of which is a disorder traditionally characterized by short stature [132, 133]. Since CENPJ regulates several aspects of centrosome function, a loss of this protein may lead to deficits in multiple cellular pathways which act together to cause dwarfism.

## 10. STIL (SCL/TAL1-Interrupting Locus)

STIL is a centrosomal protein localized to newly synthesized centrioles [118, 134, 135]. Immediately after PLK4 is recruited by CEP152 and CEP192, SAS-6 and STIL are brought to the site of centriole assembly. SAS-6 directly interacts and forms a complex with STIL, and these two proteins resemble each other in many ways in terms of functionality, subcellular localization pattern, and expression levels during the cell cycle [118, 134, 135]. Ablation of one protein causes mislocalization of the other, suggesting that STIL and SAS-6 are mutually dependent for their localization to centrioles. Moreover, upregulation of STIL induces the formation of multiple nascent centrioles around the parental centriole, mimicking the phenotype of SAS-6 or PLK4 overexpression [106, 136, 137]. STIL appears to be important for spindle positioning and mitotic progression [59, 138, 139]. Inactivation of sil in zebrafish or STIL in mice results in embryonic lethality (Table 2), indicating that this protein may play additional roles beyond brain development [139, 140].

## 11. CEP135 (Centrosomal Protein of 135 kDa)

As an important regulator of centrosome biogenesis, CEP135 is a centriolar protein that associates with SAS-6 and CENPJ [123, 141, 142]. The precise relationship between CEP135 and SAS-6 is not completely clear at this point, although these proteins do not appear to depend on each other for localization to the centriole. On the other hand, CEP135 recruits CENPJ to centrioles, and not vice versa, indicating that CEP135 likely functions upstream of CENPJ [123]. Furthermore, in contrast to the loss of PLK4, SAS-6, or STIL, which completely suppresses centriole duplication, ablation of CEP135 results in a less severe phenotype with shorter centrioles and atypical centriolar structure [123, 143]. By the same token, abnormal centrioles are also observed in* Drosophila*,* Chlamydomonas*,* Tetrahymena*, and* Paramecium cep135* mutants (Table 2), and these results collectively suggest that the structural integrity of centrioles is compromised [144–150]. Since these structural anomalies are known to induce mitotic defects, including the formation of monopolar spindles, it would be interesting in the long run to investigate their consequences on prenatal neurogenesis [123, 143].

## 12. CEP152 (Centrosomal Protein of 152 kDa)

Although CEP152 is deficient in both MCPH and SCKL, mutations in* CEP152* are by far the most common cause of SCKL, accounting for the majority of cases [27, 151]. During the early step of centriole duplication, CEP152 and its associated partner, CEP192 form a discrete ring around parental centrioles, making the site of PLK4 recruitment and nascent centriole assembly [107, 108, 152]. The centrosomal localization of CEP152 is also dependent on CEP192, but not vice versa. In addition, CEP152 is known to interact with CEP57 (centrosomal protein of 57 kDa) and CEP63 (centrosomal protein of 63 kDa), the latter of which is a SCKL protein [28, 153, 154]. While CEP152 and CEP63 are mutually dependent on one another for their centrosomal localization, only the former is absolutely required for centriole duplication, and a loss of this protein leads to severe mitotic defects and the formation of monopolar spindles [109–111]. In addition, CEP152 binds to CINP, a CDK2-interacting protein involved in DNA damage response and genome maintenance [151], thereby regulating cell cycle checkpoints. Indeed, centrosomes and nuclei show numerical and morphological abnormalities, indicative of aberrant cell division and cell cycle checkpoint, in CEP152-deficient patient fibroblasts/lymphocytes [151].

## 13. ZNF335 (Zinc Finger Protein 335)

ZNF335 is a nuclear protein and a novel component of the H3K4 methyltransferase complex involved in chromatin-remodelling and transcriptional regulation [155, 156]. One critical function of ZNF335 is its binding to the promoter region of REST/NRSF (RE1-silencing transcription factor/neuron-restrictive silencer factor), a master regulator of neuroprogenitor proliferation, and neuronal differentiation. Elegant studies using* Znf335* deficient mice have demonstrated that this protein is required for many aspects of neurodevelopment, including neurogenesis and neuronal cell fate specification, morphogenesis, and differentiation (Table 2) [156]. Perhaps because of the multifaceted nature of ZNF335, its inactivation in humans leads to a more severe phenotype compared to most reported cases of microcephaly.

## 14. PHC1 (Polyhomeotic-Like Protein 1)

PHC1 belongs to a member of the polycomb group which modulates chromatin remodelling [157]. This protein localizes to the nucleus and functions as an E3 ubiquitin ligase to promote the ubiquitination of histone H2A and to regulate the levels of GEMININ, a protein that partially localizes to the centrosome and is involved in cell cycle control [158, 159]. Depletion of PHC1 induces aberrant DNA damage repair and polyploidy, again reinforcing the view that proteins involved in cell cycle regulation and/or checkpoints are critical for brain development [157].

## 15. CDK6 (Cyclin-Dependent Kinase 6)

Hussain and coworkers have recently identified mutations in a new gene that can cause MCPH. Interestingly, this gene encodes CDK6, a well-known member of the cyclin/cyclin-dependent kinase family crucial for cell cycle progression in G1 and S phases [29, 160]. Although several previous reports have shown that CDK6 exhibits both cytoplasmic and nuclear localization during interphase, this protein, like WDR62 and ASPM, becomes enriched at the spindle poles during mitosis [29, 161–163]. Ablation of CDK6 impairs cell polarity and induces supernumerary centrosomes and aneuploidy, but it is not clear whether these phenotypes arise from cell cycle and/or spindle pole defects [29]. Interestingly,* Cdk6* knock-out mice are viable and develop normally (Table 2), suggesting that this protein is dispensable for proliferation in most cell types [164]. Paradoxically, a more recent study illustrates the importance of Cdk6 in embryonic neurogenesis and demonstrates that inactivation of* Pax6*, a transcription factor that directly represses the expression of* Cdk6*, leads to inappropriate activation of Cdk6 and overproliferation of neuroprogenitor cells in mice [165]. Another study also highlighted a role for CDK6 in the regulation of G1 length during adult neurogenesis, although its potential contribution to embryonic neurogenesis was not addressed [166].

## 16. A Multiprotein Complex in Brain Development

**Figure 2:**
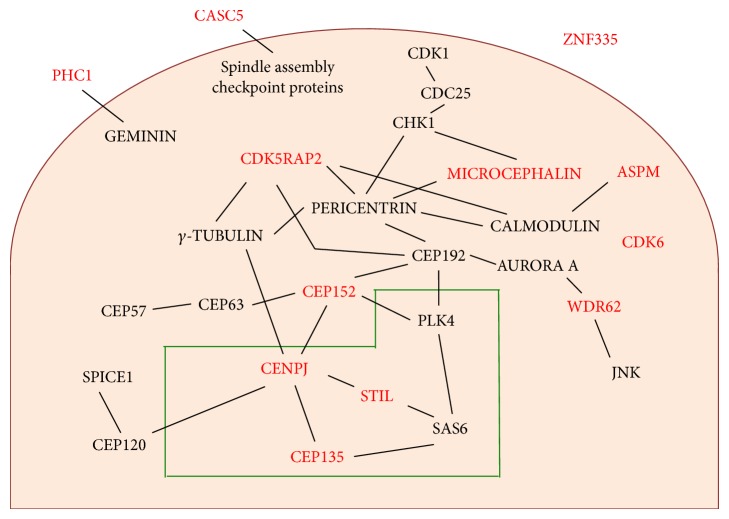
Microcephaly protein interaction network. The majority of microcephaly proteins (red) are associated with centrosomes. CENPJ, STIL, and CEP135 are components of centrioles (green box), while MICROCEPHALIN, CDK5RAP2, and CEP152 are part of the PCM (orange background). WDR62, ASPM, and CDK6 temporarily localize to the PCM. In addition, CASC5 and PHC1 are known to interact with proteins at the centrosome. For ZNF335, its precise connection to the centrosome is not understood. Microcephaly proteins are physically linked to one another either directly or indirectly (solid black lines) to form a protein network.

In summary, almost all MCPH proteins are linked to the centrosome with varying levels of intimacy (Figure 2). CENPJ, STIL, and CEP135 are core centriolar components; MICROCEPHALIN, CDK5RAP2, and CEP152 form an integral part of the PCM; WDR62, ASPM, and CDK6 are transiently associated with the centrosome; and two other microcephaly proteins, CASC5 and PHC1, interact with known centrosomal constituents. In addition, a handful of microcephaly proteins are physically linked to each other, either directly or indirectly, suggesting the existence of a large network of protein-protein interactions essential for prenatal neurogenesis. We believe that the loss of a single protein or protein-protein interaction could cripple the interaction network, thereby increasing susceptibility to disease. Tellingly, mutations in PERICENTRIN, a protein in the network that interacts with MICROCEPHALIN and CDK5RAP2, cause SCKL and primordial dwarfism [167]. Furthermore, upregulation of Plk4 is shown to impede brain development and cause microcephaly in mice, although there have been no reported cases in humans so far [168]. Moreover, CEP63 is a protein deficient in SCKL and known to interact with CEP152 [28]. As additional disease genes are being rapidly discovered, it is intriguing to speculate on their identity and whether they encode proteins in the interaction network.

## 17. Conclusion

**Figure 3:**
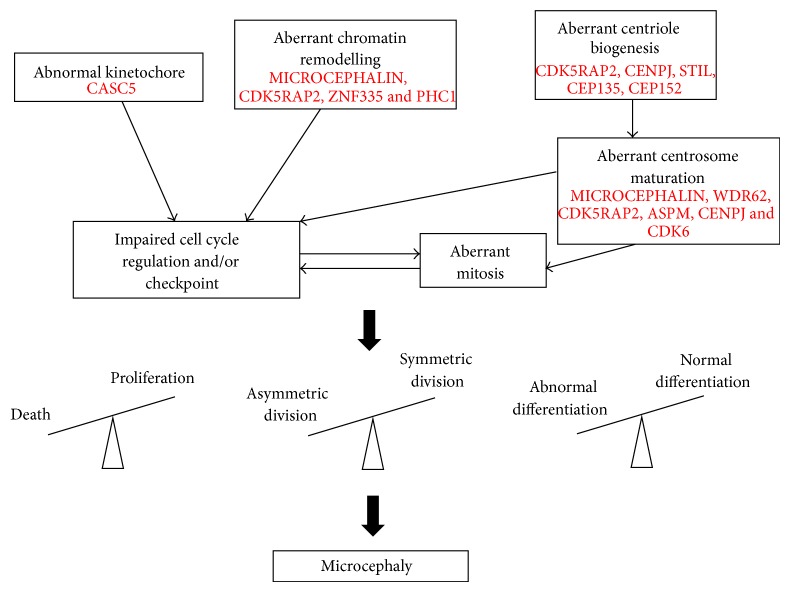
Cellular processes involved in microcephaly. A model depicting how malfunction of microcephaly proteins perturbs neurogenesis. A loss of microcephaly proteins can disturb various cellular processes, including chromatin remodelling, kinetochore integrity, centrosome biogenesis, or centrosome maturation, which impair cell cycle checkpoints and mitosis. These perturbations disrupt the equilibrium between cell proliferation and cell death, symmetric and asymmetric division, and/or normal and abnormal differentiation, reducing the total number of neuroprogenitor cells and differentiated neurons in the developing brain, leading to microcephaly.

During the past decade, substantial progress has been made in our understanding of brain development and the genetic basis of MCPH. It is now apparent that microcephaly proteins control a number of cellular processes, including centriole biogenesis, centrosome maturation, cell cycle and DNA damage checkpoint, spindle positioning, and mitosis, all of which impinge on brain growth and size (Figure 3). While neuronal homeostasis is thought to be maintained by a complex interplay between the opposing actions of cell proliferation and cell death, symmetric and asymmetric division, and/or normal and aberrant differentiation, to what extent does each of these contribute to brain development? Despite our knowledge of microcephaly proteins, many important questions remain. For instance, why do some proteins appear to have a better-defined role in regulating the switch between symmetric and asymmetric division in the developing brain, and why others are more frequently involved in SCKL and/or have additional functions in more than one cell/tissue type? Is the centrosome a central hub for coordinating and integrating various molecular events crucial for prenatal neurogenesis? We firmly believe that the answers to these questions hinge on our ability to fully understand the functional importance of each microcephaly protein and build upon the existing protein interaction network. These studies would help to better define microcephaly disorders and to facilitate early diagnosis and prognosis.

## Figures and Tables

**Table 1:** Gene table: autosomal recessive primary microcephaly (MCPH).

Gene	Locus	Gene product	References (gene and/or locus)	OMIM
*MCPH1 MICROCEPHALIN *	MCPH1	MICROCEPHALIN	[40]	607117
*WDR62 (WD repeat-containing protein 62) *	MCPH2	WDR62	[169]	613583
*CDK5RAP2 (CDK5 regulatory subunit-associated protein 2) *	MCPH3	CDK5RAP2	[170]	608201
*CASC5 (cancer susceptibility candidate 5) *	MCPH4	CASC5	[171]	609173
*ASPM (abnormal spindle-like microcephaly-associated protein) *	MCPH5	ASPM	[172]	605481
*CENPJ (centromere protein J) *	MCPH6	CENPJ	[15]	609279
*STIL (SCL/TAL1-interrupting locus) *	MCPH7	STIL	[25]	181590
*CEP135 (centrosomal protein of 135 kDa) *	MCPH8	CEP135	[26]	611423
*CEP152 (centrosomal protein of 152 kDa) *	MCPH9	CEP152	[27]	613529
*ZNF335 (zinc finger protein 335) *	MCPH10	ZNF335	[156]	610827
*PHC1 (polyhomeotic-like protein 1) *	MCPH11	PHC1	[157]	602978
*CDK6 (cyclin-dependent kinase 6) *		CDK6	[29]	603368

**Table 2:** Animal models of MCPH.

Gene	Model	Method	Phenotype
*MCPH1 MICROCEPHALIN *	Mouse	Knock-out (deletion of exon 2)	Genomic instability, growth retardation, male infertility, and increased radiation sensitivity
	Mouse	Knock-out (gene trap)	Shorter life span, improper chromosome condensation
	Mouse	Conditional knock-out (recombination)	Specific reduction of the cerebral cortex at birth
	Fly	Knock-out (p-element excision)	Abnormal spindles during embryonic cell cycle

*WDR62 *	Rat	shRNA knock-down	Premature differentiation of neuroprogenitors into neurons
	Zebrafish	Morpholino-mediated knock-down	Reduction in head and eye size
	Mouse	Knock-out (deletion of the WDR62 locus)	Reduced brain size

*CDK5RAP2 *	Fly	Knock-out (chemical mutagenesis)	Disconnection between centrosome and PCM
	Mouse	shRNA knock-down	Premature neuronal differentiation
	*Hertwig's anemia* mouse	Inversion of exon 4	Reduced brain size

*ASPM *	Zebrafish	Morpholino-mediated knock-down	Reduction in head and eye size
	Mouse	siRNA knock-down	Premature differentiation of telencephalic neuroprogenitor cells
	Mouse	Knock-out (removal of exons 2 and 3)	Reduced brain size
	Mouse	Knock-out (gene trap)	Mild microcephaly, massive loss of germ cells
	Fly	Mutagenesis (x-irradiation)	Spindle positioning defects, increased apoptosis

*CENPJ *	Mouse	Conditional knock-out (truncated mRNA)	Intrauterine growth retardation
	Fly	Knock-out (transposon insertion)	Loss of centrioles, abnormal spindle
	Worm	siRNA knock-down	Loss of centrioles, abnormal centrosome size/organization

*STIL *	Mouse	Knock-out (removal of exons 3 to 5)	Embryonic lethality
	Zebrafish	Morpholino-mediated knock-down	Embryonic lethality

*CEP135 *	Fly	Knock-out (transposon insertion)	Abnormal centrioles, immotile cilium
	Alga	Insertion mutagenesis	Abnormal centrioles, abnormal cell division, and slow growth
	Protozoa	siRNA knock-down	Abnormal centrioles

*CEP152 *	Fly	Chemical mutagenesis	Defective centrosomes, no zygotic division
	Zebrafish	Morpholino-mediated knock-down	Ciliary defects

*ZNF335 *	Mouse	shRNA knock-down	Impaired progenitor cell proliferation
	Mouse	Knock-out (removal of promoter and exons 1 and 2)	Severely reduced cortical size
	Mouse	Knock-out (gene trap insertion)	Embryonically lethal

*CDK6 *	Mouse	Knock-out (removal of 1st coding exon)	Viable, develop normally, hematopoiesis slightly impaired
